# Joint Beamforming Design for Active RIS-Assisted ISAC Systems with Transmitter Hardware Impairments

**DOI:** 10.3390/s26154682

**Published:** 2026-07-23

**Authors:** Zhen Li, Jinhui Hu, Jian Xing

**Affiliations:** College of Control and Information Engineering, Northeast Forestry University, Harbin 150040, China; lz5301@nefu.edu.cn (Z.L.); hujinhui@nefu.edu.cn (J.H.)

**Keywords:** active RIS, integrated sensing and communication, hardware impairments, joint beamforming optimization

## Abstract

This paper investigates an active reconfigurable intelligent surface (RIS)-assisted integrated sensing and communication (ISAC) system with transmitter hardware impairments (HWIs) at the base station (BS). The active RIS provides both phase adjustment and amplitude amplification, which helps mitigate the multiplicative fading effect of passive RIS-assisted cascaded links and establish virtual line-of-sight (LoS) links for the target and multiuser communication users. Considering the coupling among the BS transmitter distortion noise, active RIS amplification noise, and the active RIS power constraint, we formulate a radar output signal-to-noise ratio (SNR) maximization problem. The radar output SNR is maximized by jointly designing the radar receive filter, the BS transmit beamforming matrix, and the active RIS reflection coefficients, while satisfying the quality-of-service (QoS) requirements of communication users, the BS transmit power constraint, and the active RIS power budget constraint. To solve the resulting non-convex problem with fractional objectives and high-order coupling terms, an alternating optimization (AO)-based iterative framework is developed. Specifically, the radar receive filter is updated using the generalized Rayleigh quotient, the BS transmit beamforming subproblem is handled by semidefinite relaxation and the Charnes–Cooper transformation, and the active RIS reflection coefficient subproblem is solved using the Dinkelbach transformation and majorization–minimization. Simulation results demonstrate stable convergence and show that the proposed design improves radar output SNR under BS transmitter HWIs.

## 1. Introduction

The development of sixth-generation (6G) mobile communication systems is expected to place higher demands on spectral efficiency (SE), hardware resource reuse, and environmental sensing capabilities in future intelligent wireless networks [1,2]. Integrated sensing and communication (ISAC) enables information transmission and target sensing in a coordinated manner by sharing spectrum resources, hardware platforms, and signal processing frameworks. Therefore, it has the potential to improve system resource utilization efficiency and support typical applications, such as intelligent transportation, smart cities, and the Industrial Internet of Things [3,4,5]. However, practical base station (BS) transmitters are subject to hardware impairments (HWIs) arising from the non-ideal characteristics of radio-frequency (RF) components [6]. Specifically, the BS transmit RF chains may suffer from power-amplifier nonlinearities, local-oscillator phase noise, in-phase/quadrature-phase (I/Q) imbalance, digital-to-analog converter (DAC) quantization errors, and amplitude or phase mismatches among RF chains [7]. These BS transmitter HWIs may introduce residual distortion into the transmitted ISAC waveform, thereby affecting both the communication signals received by the users and the radar echoes received at the BS [8,9]. Therefore, explicitly considering BS transmitter HWIs in joint beamforming design is important for accurately evaluating the communication and sensing performance of practical ISAC systems.

For ISAC systems operating under non-ideal hardware conditions, existing studies have incorporated HWIs into joint communication and sensing beamforming design. Reference [8] investigated joint radar-communication beamforming design by considering both transceiver HWIs and imperfect channel state information. In reference [9], beamforming optimization was further studied for multiuser and multitarget ISAC scenarios in the presence of transceiver HWIs. These studies indicate that explicitly modeling hardware non-idealities is important for the design of practical ISAC systems. However, in practical deployments, severe blockages and path loss still degrade both communication and radar echo links [10,11]. Therefore, in addition to considering RF hardware non-idealities, improving the accessibility of communication and sensing links in complex propagation environments remains an issue that needs to be further addressed in practical ISAC system design.

To improve the availability and reliability of communication and sensing links in complex propagation environments, the reconfigurable intelligent surface (RIS) has emerged as a promising technology for ISAC systems by reconfiguring the wireless electromagnetic environment. By controlling the reflection of incident electromagnetic waves, RIS can establish controllable reflected links, thereby improving the link quality of communication users and sensing targets in blockage scenarios [12,13]. For passive RIS-assisted ISAC systems, existing studies have investigated joint transmit beamforming and reflection phase-shift design, communication quality-of-service (QoS) constraints, multiuser and multitarget scenarios, clutter suppression, and signal-to-noise ratio (SNR)- and Cramér–Rao bound (CRB)-constrained optimization [14,15,16,17,18,19,20,21]. These studies indicate that passive RIS can provide additional spatial degrees of freedom for ISAC systems and improve communication and sensing performance under complex propagation conditions. However, due to the passive reflection mechanism, passive RIS mainly adjusts the signal propagation direction through phase control, while the Tx–RIS–Rx cascaded link still suffers from multiplicative fading and the double path-loss effect [22]. Therefore, in scenarios where the loss of obstructed cascaded links is relatively pronounced, the introduction of active RIS with amplitude amplification capability provides a reasonable research direction.

To alleviate the path-loss issue in passive RIS-assisted cascaded links, an active RIS integrates power amplifiers into its reflecting elements to achieve amplitude enhancement while regulating the phase of the reflected signals, thereby increasing the equivalent received signal power of the cascaded links [23,24,25]. Motivated by these characteristics, active RISs have been increasingly incorporated into ISAC system research. References [26,27,28] investigated the joint optimization of the BS transmit beamforming matrix, active RIS reflection coefficients, and communication and sensing performance metrics. Reference [29] investigated physical-layer security design in active RIS-assisted ISAC systems. In [30], the authors investigated active RIS-assisted target parameter estimation based on the CRB from the perspective of theoretical sensing performance bounds.

Beyond the above studies based on ideal RIS models, practical hardware non-idealities in RIS- and active-RIS-assisted systems have also attracted increasing research attention. At the RIS element level, practical reflection coefficients may exhibit phase-dependent amplitude responses. Such amplitude–phase coupling has been incorporated into the robust beamforming design for RIS-assisted MIMO communication systems with imperfect cascaded channel state information (CSI) [31]. Finite-resolution phase control and the resulting phase quantization represent another major category of practical RIS-side hardware limitations. Such element-level phase quantization has been widely considered in standardization-focused system-level simulations of multi-RIS and multi-BS networks, where downlink reference signal received power (RSRP) and signal-to-interference-plus-noise ratio (SINR) are taken as the primary performance metrics. Further system-level assessments also clarify the impact of practical RIS deployment on system capacity and cell coverage [32]. Non-uniform phase quantization and heterogeneous phase-resolution configurations have also been studied in RIS-assisted communication systems, with their impacts on the achievable received signal power and the transmit power required to meet a prescribed minimum SNR constraint fully characterized [33]. In RIS-assisted MIMO communication systems, the joint effects of transceiver HWIs, RIS phase noise, and imperfect CSI have been investigated via robust transceiver and reflection-matrix designs [34]. Transceiver HWIs and active RIS phase noise have also been jointly addressed in active RIS-assisted multiuser communications, where the BS transmit beamforming, amplification factors, and phase shifts were optimized to maximize the sum-rate [35]. Moreover, the nonlinear distortion generated by active RIS amplifiers and its spatial radiation characteristics have been investigated [36]. Non-ideal RIS responses have also been considered in RIS-assisted ISAC systems, where the transmit beamforming and RIS reflection coefficients were jointly designed for short-packet communication and sensing [37]. Collectively, these studies provide a broader understanding of passive RIS and active RIS hardware non-idealities in communication and ISAC systems.

Despite the progress summarized above, active RIS-assisted ISAC design in the presence of residual BS transmitter HWIs remains relatively less explored. In such systems, the distortion noise generated at the BS transmitter propagates through both the communication links and the active RIS-assisted round-trip sensing link, thereby affecting the received communication signals and radar echoes. Meanwhile, the active RIS amplification noise and reflection power consumption are closely coupled with the active RIS reflection coefficients. Consequently, the resulting joint beamforming optimization problem involves a fractional objective function, highly coupled variables, and multiple non-convex constraints. Reference [38] investigated active RIS-assisted ISAC beamforming design in the presence of BS transmitter HWIs from a deep reinforcement learning (DRL) perspective. The authors employed the MPC-TD3 method to jointly optimize the BS transmit beamforming matrix and the active RIS reflection coefficients, providing a data-driven solution for such non-convex problems. Distinct from the aforementioned policy-learning-based approach, this paper proceeds from the mathematical structure of the original optimization problem and explicitly models the coupling relationships among transmitter distortion noise, active RIS amplification noise, the active RIS power constraint, and communication QoS constraints. By integrating optimization tools such as variable decoupling, convex relaxation, fractional programming transformations, and iterative approximation, this paper provides a structured joint beamforming optimization framework for the considered problem.

Based on the above analysis, this paper further investigates the joint beamforming optimization problem for an active RIS-assisted ISAC system in the presence of BS transmitter HWIs. Different from existing DRL-based methods that mainly obtain approximate beamforming decisions through policy learning, this paper starts from the mathematical structure of the original optimization problem. Specifically, the radar receive filter, the BS transmit beamforming matrix, and the active RIS reflection coefficients are jointly optimized to maximize the radar output SNR, subject to multiuser communication QoS requirements, the BS transmit power constraint, and the active RIS power budget constraint. Specifically, the BS transmitter distortion noise is signal-dependent, and its covariance is determined by the BS transmit beamforming matrix. Meanwhile, the active RIS amplification noise and active reflection power are closely coupled with the reflection coefficients and cascaded channels. As a result, the formulated problem involves a fractional objective function, high-order coupling terms, and multiple non-convex constraints. To address these challenges, this paper proposes an iterative solution framework based on alternating optimization and convexification. By using optimization tools such as variable decomposition, convex relaxation, fractional programming transformations, and iterative approximation, the proposed framework explicitly handles the coupling structures in the original problem and enables an iterative solution procedure. The main contributions of this paper are summarized as follows:

- We formulate a joint beamforming optimization problem for an active RIS-assisted multiuser ISAC system under BS transmitter HWIs. The considered system consists of a multi-antenna BS, multiple single-antenna communication users, an active RIS, and a sensing target. In the proposed model, the residual distortion noise caused by BS transmitter HWIs depends on the BS transmit beamforming matrix and propagates through the direct communication links, the RIS-assisted communication links, and the round-trip radar echo links. Moreover, the coupling among the active RIS amplification noise, reflection coefficients, and RIS power consumption is explicitly characterized. Based on this model, a radar output SNR maximization problem is formulated by jointly considering the communication QoS constraints, the BS transmit power constraint, and the active RIS power budget.
- We propose an alternating optimization (AO)-based algorithm to address the coupling among the distortion noise induced by BS transmitter HWIs, the active RIS amplification noise, and the fractional radar output SNR objective. The original non-convex problem is decomposed into three iterative subproblems. For the radar receive filter, a closed-form solution is obtained based on the generalized Rayleigh quotient. For the BS transmit beamforming matrix, semidefinite relaxation (SDR) and the Charnes–Cooper transformation are employed to convert the fractional beamforming subproblem into a tractable semidefinite programming (SDP) problem. For the active RIS reflection coefficients, the Dinkelbach transformation is used to handle the fractional objective, while the majorization–minimization (MM) method is applied to construct quadratic surrogate functions for the quartic coupling terms. In this way, the original highly non-convex problem is solved through a sequence of tractable convex subproblems, achieving joint optimization with theoretically guaranteed convergence.
- We evaluate the proposed joint beamforming design under different levels of BS transmitter HWIs through numerical simulations. Under the considered ideal-CSI simulation settings, the proposed AO-based algorithm exhibits stable numerical convergence and typically converges within approximately 10 iterations. For the baseline blocked-direct-link configuration and the adopted system parameters, the active RIS-assisted ISAC scheme achieves an approximately 30dB radar output SNR improvement over the passive RIS-assisted benchmark. Moreover, within the considered range of the BS transmitter HWI coefficient, the proposed joint design maintains a higher radar output SNR than the baseline schemes, illustrating the benefit of jointly optimizing the BS transmit beamforming matrix, active RIS reflection coefficients, and radar receive filter. The effects of key system parameters, including the BS transmit power, array sizes, RIS deployment position, Rician factor, active RIS power budget, and communication QoS threshold, are also investigated.

The remainder of this paper is organized as follows. Section 2 presents the system model and formulates the joint radar receive filtering, BS transmit beamforming, and active RIS reflection design problem. Section 3 develops the proposed AO-based joint beamforming and reflection design framework and analyzes its computational complexity. Section 4 presents the numerical simulation results and discusses the effects of key system parameters on the radar output SNR. Finally, Section 5 concludes the paper.

*Notations:* Boldface lowercase and uppercase letters denote vectors and matrices, respectively. C represents the set of complex numbers. ${\left(\cdot \right)}^{T}$, ${\left(\cdot \right)}^{H}$, ${\left(\cdot \right)}^{*}$, and ${\left(\cdot \right)}^{-1}$ denote the transpose, conjugate transpose, complex conjugate, and matrix inverse, respectively. Tr(·), rank{·}, and vec{·} denote the trace, rank, and vectorization operations, respectively. diag{·} constructs a diagonal matrix whose diagonal entries are given by the vector a, ${∥\cdot ∥}_{2}$ denotes the Euclidean norm and ⊗ denotes the Kronecker product. $I_{N}$ denotes the N×N identity matrix. E{·}, Re{·}, and Im{·} denote the expectation, real-part, and imaginary-part operators, respectively. O(·) denotes the asymptotic computational complexity.

## 2. System Model and Problem Formulation

**Figure 1:**
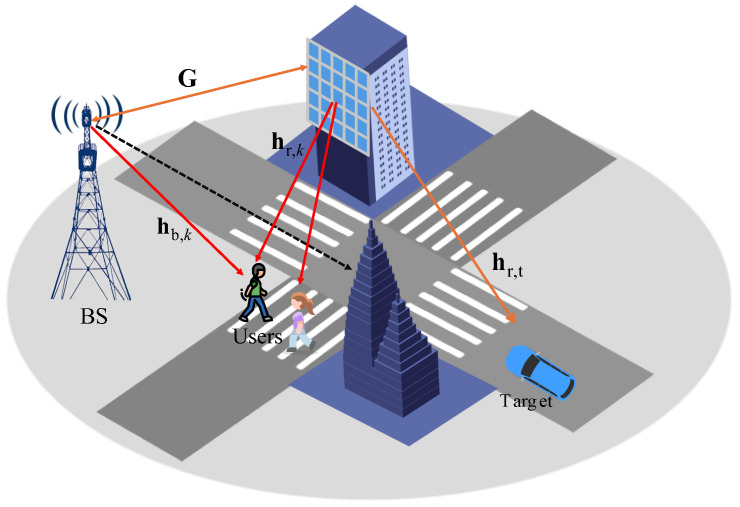
An active RIS-assisted ISAC system.

This paper considers an active RIS-assisted ISAC system, as shown in Figure 1. The system consists of a BS equipped with *N* transmit/receive antennas, *K* single-antenna communication users, an active RIS with *M* reflecting elements, and a sensing target. The BS operates in full-duplex (FD) mode to support simultaneous signal transmission and radar echo reception. To focus on the effects of BS transmitter HWIs and their interactions with the active RIS amplification process and power constraint, residual self-interference (RSI) after self-interference cancellation (SIC) is assumed to be negligible. This idealized assumption simplifies the radar receive model but constitutes an important limitation of the present study, since non-negligible RSI may remain in practical FD implementations and degrade radar sensing performance. The target is located in a region blocked from the BS; thus, the sensing task relies on the active RIS-assisted BS–RIS–target–RIS–BS round-trip reflected link. The reflecting elements of the active RIS support joint phase control and signal amplification through built-in amplifier circuits, thereby alleviating the pronounced double path-loss effect in RIS-assisted cascaded links.

Perfect global channel state information (CSI) is assumed to be available at the BS. Accordingly, the system formulation and the proposed AO-based algorithm are developed as an ideal-CSI benchmark. This assumption is adopted to maintain a focused analysis of the effects of BS transmitter HWIs and the coupling of BS transmitter distortion noise with the active RIS amplification noise and power constraint. Incorporating imperfect CSI would require an explicit bounded or stochastic channel-uncertainty model and a corresponding robust reformulation of the communication QoS constraints, the radar output SNR expression, and the active RIS power constraint, which is beyond the scope of the present study.

### 2.1. Signal Transmission Model

To characterize the impact of transmitter HWIs on the BS transmit signal, residual distortion noise is introduced into the ideal ISAC transmit signal. Accordingly, the actual transmit signal at the BS can be expressed as

(1) $$x=W_{c}s_{c}+W_{r}s_{r}+n_{b}=Ws+n_{b},$$

where $s_{c}\in C^{K\times 1}$ denotes the communication symbol vector intended for the *K* users satisfying $E\left\{s_{c}s_{c}^{H}\right\}=I_{K}$, and $s_{r}\in C^{N\times 1}$ denotes the *N* independent radar signals satisfying $E\left\{s_{r}s_{r}^{H}\right\}=I_{N}$, and the communication signal and radar signals are assumed to be statistically independent and uncorrelated, i.e., $E\{s_{c}s_{r}^{H}\}=0$. For notational simplicity, the joint transmit beamforming matrix and the stacked signal vector are respectively defined as $W≜\left[W_{c},W_{r}\right]\in C^{N\times (N+K)}$ and $s≜{\left[s_{c}^{T},s_{r}^{T}\right]}^{T}\in C^{(N+K)\times 1}$, where $W_{c}\in C^{N\times K}$ and $W_{r}\in C^{N\times N}$ denote the corresponding beamforming matrices for communication symbols and radar signals, respectively. $n_{b}∼\mathrm{CN}\left(0,\lambda \mathrm{diag}\left\{WW^{H}\right\}\right)$ represents the additive distortion noise introduced by BS transmitter HWIs, where λ∈(0,1) is the hardware impairment coefficient. Accordingly, the covariance matrix of the distortion noise is defined as $R_{b}≜\lambda \mathrm{diag}\left\{WW^{H}\right\}$. Thus, the transmit power $P_{\mathrm{BS}}$ at the BS is given by

(2) $$P_{\mathrm{BS}}=\mathrm{Tr}\left(R_{x}\right),$$

where $R_{x}≜WW^{H}+R_{b}$ denotes the covariance matrix of the actual transmit signal at the BS.

### 2.2. Communication Model

The received signal at the *k*-th communication user consists of the signals from the direct link and the active RIS-assisted cascaded link, the amplification noise introduced by the active RIS, and the receiver background thermal noise, which can be calculated as

(3) $$y_{k}=\left(h_{b,k}^{H}+h_{r,k}^{H}\Phi G\right)x+h_{r,k}^{H}\Phi n_{0}+n_{k},$$

where $G\in C^{M\times N}$, $h_{b,k}\in C^{N\times 1}$, and $h_{r,k}\in C^{M\times 1}$ denote the BS–RIS channel, the BS–user channel, and the RIS–user channel associated with the *k*-th user, respectively. The reflection coefficient matrix of the active RIS is defined as $\Phi ≜\mathrm{diag}\;\left(\phi \right)$ where $\phi ≜{\left[\phi_{1},\ldots ,\phi_{M}\right]}^{T}$. Different from a passive RIS, an active RIS integrates power amplifiers into its reflecting elements, thereby enabling joint phase control and amplitude amplification of the reflected signals at the element level. The *m*-th reflection coefficient is expressed as $\phi_{m}≜a_{m}e^{j\varphi_{m}}$, where $a_{m}\in \left(0,a_{\max}\right]$ and $\varphi_{m}\in \left[0,2\pi \right)$ denote the reflection amplitude and phase shift, respectively. For analytical tractability and to establish an ideal performance benchmark, continuous and independent amplitude and phase control is assumed, while practical finite-resolution control and circuit-dependent amplitude–phase coupling are beyond the scope of the present study. Furthermore, let $n_{0}∼\mathrm{CN}\left(0,\sigma_{s}^{2}I_{M}\right)$ and $n_{k}∼\mathrm{CN}\left(0,\sigma_{k}^{2}\right)$ denote the dynamic thermal noise introduced by the active RIS and the additive white Gaussian noise (AWGN) at the *k*-th user, respectively. Accordingly, the SINR of the *k*-th user is given by

(4) $$\gamma_{k}=\frac{{\left|h_{k}^{H}w_{k}\right|}^{2}}{\sum_{i\neq k}^{K+N}{\left|h_{k}^{H}w_{i}\right|}^{2}+h_{k}^{H}R_{b}h_{k}+{∥h_{r,k}^{H}\Phi ∥}^{2}\sigma_{s}^{2}+\sigma_{k}^{2}},$$

where $h_{k}^{H}≜h_{b,k}^{H}+h_{r,k}^{H}\Phi G$ denotes the equivalent cascaded channel from the BS to the *k*-th user, and $w_{i}$ denotes the *i*-th column of the BS transmit beamforming matrix $W≜[w_{1},\ldots ,w_{K+N}]$.

### 2.3. Radar Signal Model

Since the direct BS–target link is blocked, we assume that the radar echo mainly propagates through the active RIS-assisted BS–RIS–target–RIS–BS reflected link. The radar echo signal received at the BS can be written as

(5) $$y_{r}=G^{H}\Phi^{H}\left[\beta h_{r,t}h_{r,t}^{H}\Phi \left(Gx+n_{0}\right)+n_{1}\right]+n_{r},$$

where $\beta ∼\mathrm{CN}(0,\sigma_{t}^{2})$ denotes the radar cross section (RCS), and $h_{r,t}\in C^{M\times 1}$ denotes the LoS channel between the RIS and the target. The target angle of arrival (AoA) and angle of departure (AoD) with respect to the RIS are assumed to be obtained from a preceding target acquisition or tracking stage and are treated as known during the subsequent beamforming design. In practice, low-complexity AoA estimation techniques, such as the 1-bit conversion-based method in [39], may provide a possible approach for obtaining the arrival-angle information. In the present formulation, perfect angular information is assumed, while angular estimation errors may cause steering-vector mismatch and degrade the radar output SNR. Furthermore, $n_{1}∼\mathrm{CN}\left(0,\sigma_{s}^{2}I_{M}\right)$ denotes the dynamic thermal noise introduced by the active RIS during the echo-signal amplification phase. It is assumed to be independent of $n_{0}$ and to follow the same distribution. In addition, $n_{r}∼\mathrm{CN}\left(0,\sigma_{r}^{2}I_{N}\right)$ denotes the AWGN at the BS receiver. After processing the received signal $y_{r}$ with a receive filter $u\in C^{N\times 1}$, the radar output signal at the BS is written as

(6) $$u^{H}y_{r}=\beta u^{H}Ax+\beta u^{H}Bn_{0}+u^{H}Pn_{1}+u^{H}n_{r},$$

where the auxiliary matrices A, B, and P are defined as

(7) $$\begin{array}{c} A≜G^{H}\Phi^{H}h_{r,t}h_{r,t}^{H}\Phi G, \end{array}$$

(8) $$\begin{array}{c} B≜G^{H}\Phi^{H}h_{r,t}h_{r,t}^{H}\Phi , \end{array}$$

(9) $$\begin{array}{c} P≜G^{H}\Phi^{H}. \end{array}$$

Accordingly, the radar output SNR is formulated as

(10) $$\gamma_{r}=\frac{\sigma_{t}^{2}u^{H}AWW^{H}A^{H}u}{u^{H}\left(\sigma_{t}^{2}AR_{b}A^{H}+\sigma_{t}^{2}\sigma_{s}^{2}BB^{H}+\sigma_{s}^{2}PP^{H}+\sigma_{r}^{2}I_{N}\right)u}.$$

It should be noted that the radar output SNR in Equation (10) is derived under the assumption that RSI after SIC is negligible. Under imperfect SIC, the RSI covariance would contribute an additional term to the interference-plus-noise covariance in the denominator of the radar output SNR, thereby degrading the sensing performance and potentially introducing further coupling between the BS transmit beamforming matrix and the radar receive filter. Incorporating RSI into the joint optimization framework is left for future work.

### 2.4. Problem Formulation

Based on the above system model, this paper formulates a joint beamforming optimization problem, where the optimization variables include the BS transmit beamforming matrix W, the active RIS reflection coefficient vector ϕ, and the radar receive filter u. The objective is to maximize the radar output SNR, subject to the QoS threshold $\Gamma_{k}$ of each communication user, the transmit power budget $P_{\mathrm{BS}}$ at the BS, the power budget $P_{\mathrm{RIS}}$ at active RIS, and the reflection amplitude upper bound $a_{\max}$ of the active RIS. Therefore, the optimization problem can be formulated as

(11) $$\begin{array}{cc} \max_{u,W,\phi }\;\; & \gamma_{r} \end{array}$$

(12) $$\begin{array}{cc} s.t.\;\; & \mathrm{Tr}\left(R_{x}\right)\leq P_{\mathrm{BS}}, \end{array}$$

(13) $$\begin{array}{cc} \; & \gamma_{k}\geq \Gamma_{k},\;\forall k, \end{array}$$

(14) $$\begin{array}{cc} \; & P_{\mathrm{ris}}\leq P_{\mathrm{RIS}}, \end{array}$$

(15) $$\begin{array}{cc} \; & a_{m}\leq a_{\max},\;\forall m. \end{array}$$

where $P_{\mathrm{ris}}$ denotes the reflection power consumption at the active RIS and is expressed as

(16) $$\begin{array}{cc} P_{\mathrm{ris}}= & \;\sigma_{t}^{2}\mathrm{Tr}\left(\Phi^{H}h_{r,t}h_{r,t}^{H}\Phi GR_{x}G^{H}\Phi^{H}h_{r,t}h_{r,t}^{H}\Phi \right)\\ \; & +\sigma_{t}^{2}\sigma_{s}^{2}\mathrm{Tr}\left(\Phi^{H}h_{r,t}h_{r,t}^{H}\Phi \Phi^{H}h_{r,t}h_{r,t}^{H}\Phi \right)\\ \; & +\mathrm{Tr}\left(\Phi GR_{x}G^{H}\Phi^{H}\right)+2\sigma_{s}^{2}\mathrm{Tr}\left(\Phi \Phi^{H}\right). \end{array}$$

The optimization problem formulated in Equations (11)–(15) is non-convex due to the coupling among the BS transmit beamforming matrix, the active RIS reflection coefficients, and the radar receive filter. In particular, the cascaded sensing link introduces quartic terms in the radar SNR objective function in Equation (11) and the active RIS power constraint in Equation (14). Moreover, the objective function in Equation (11) and the QoS constraints in Equation (13) contain fractional structures with coupled optimization variables in the denominators. These features make the direct solution of Equations (11)–(15) non-trivial. To address these issues, this paper proposes an alternating optimization (AO)-based iterative framework, where the original non-convex problem is decomposed into a sequence of more tractable subproblems.

## 3. Joint Beamforming and Reflection Design

The original problem is difficult to solve directly due to the complicated constraints and the high-order coupling among the optimization variables W, ϕ, and u. Specifically, the distortion noise induced by BS transmitter HWIs depends on the BS transmit beamforming matrix and propagates through the active RIS-assisted cascaded link, thereby affecting both the received communication signals and the radar echo signals. This further increases the non-convexity of the problem. To address this issue, this paper proposes an AO framework that decomposes the original problem into three subproblems, namely, radar receive filter design, BS transmit beamforming design, and active RIS reflection coefficient design. The radar receive filter subproblem is solved based on the generalized Rayleigh quotient criterion. The BS transmit beamforming subproblem is handled by using SDR together with the Charnes–Cooper transformation. The active RIS reflection coefficient subproblem is solved by combining the Dinkelbach transformation and the MM method. By iteratively updating these variables, a feasible solution to the original problem is obtained.

### 3.1. Receive Filter Design

Given the BS transmit beamforming matrix W and the active RIS reflection coefficient vector ϕ, the receive-filter optimization problem can be formulated as

(17) $$\max_{u}\;\frac{\sigma_{t}^{2}u^{H}R_{s}u}{u^{H}R_{n}u}$$

where $R_{s}$ and $R_{n}$ are defined as

(18) $$\begin{array}{c} R_{s}=AWW^{H}A^{H}, \end{array}$$

(19) $$\begin{array}{c} R_{n}=\sigma_{t}^{2}AR_{b}A^{H}+\sigma_{t}^{2}\sigma_{s}^{2}BB^{H}+\sigma_{s}^{2}PP^{H}+\sigma_{r}^{2}I_{N}. \end{array}$$

The objective function formulated in Equation (17) takes the standard form of a generalized Rayleigh quotient [16]. Therefore, the optimal receive filter $u^{⋆}$ is given by the eigenvector associated with the largest eigenvalue of $R_{n}^{-1}R_{s}$ [27].

### 3.2. Transmit Beamforming Design

For fixed radar receive filter u and active RIS reflection coefficient vector ϕ, the transmit beamforming matrix W is updated by solving the following subproblem

(20) $$\begin{array}{cc} \max_{W}\;\; & \frac{\sigma_{t}^{2}u^{H}AWW^{H}A^{H}u}{u^{H}\sigma_{t}^{2}AR_{b}A^{H}u+c_{u}} \end{array}$$

(21) $$\begin{array}{cc} s.t.\;\; & \mathrm{Tr}\left(R_{x}\right)\leq P_{\mathrm{BS}}, \end{array}$$

(22) $$\begin{array}{cc} \; & \left(1+\Gamma_{k}^{-1}\right){\left|h_{k}^{H}w_{k}\right|}^{2}\geq \sum_{i=1}^{K+N}{\left|h_{k}^{H}w_{i}\right|}^{2}+h_{k}^{H}R_{b}h_{k}+c_{k},\\ \; & \sigma_{t}^{2}\mathrm{Tr}\left(\Phi^{H}h_{r,t}h_{r,t}^{H}\Phi GR_{x}G^{H}\Phi^{H}h_{r,t}h_{r,t}^{H}\Phi \right) \end{array}$$

(23) $$\begin{array}{cc} \; & \;+\mathrm{Tr}\left(\Phi GR_{x}G^{H}\Phi^{H}\right)\leq P_{\mathrm{RIS}}-c_{p}, \end{array}$$

where $c_{u}$, $c_{k}$, and $c_{p}$ collect the terms independent of the BS transmit beamforming matrix W and are given by

(24) $$\begin{array}{c} c_{u}≜u^{H}\left(\sigma_{t}^{2}\sigma_{s}^{2}BB^{H}+\sigma_{s}^{2}PP^{H}+\sigma_{r}^{2}I_{N}\right)u, \end{array}$$

(25) $$\begin{array}{c} c_{k}≜{∥h_{r,k}^{H}\Phi ∥}^{2}\sigma_{s}^{2}+\sigma_{k}^{2}, \end{array}$$

(26) $$\begin{array}{c} c_{p}≜\sigma_{t}^{2}\sigma_{s}^{2}\mathrm{Tr}\left(\Phi^{H}h_{r,t}h_{r,t}^{H}\Phi \Phi^{H}h_{r,t}h_{r,t}^{H}\Phi \right)+2\sigma_{s}^{2}\mathrm{Tr}\left(\Phi \Phi^{H}\right). \end{array}$$

The presence of multiuser interference (MUI) terms and the quadratic forms in both the objective function and constraints render the problem formulated in Equations (20)–(23) non-convex. To facilitate the subsequent SDR reformulation, the following matrix lifting variables are introduced:

(27) $$\begin{array}{c} Q_{i}≜w_{i}w_{i}^{H}⪰0,\;i=1,\ldots ,K, \end{array}$$

(28) $$\begin{array}{cc} S & ≜W_{r}W_{r}^{H}+\sum_{i=1}^{K}Q_{i}=WW^{H}, \end{array}$$

where $Q_{i}$ and S are Hermitian positive semidefinite matrices satisfying $Q_{i}⪰0$, S⪰0 and $\mathrm{rank}\{Q_{i}\}=1,\forall i$. The matrix $S-\sum_{i=1}^{K}Q_{i}⪰0$ represents the covariance matrix of the dedicated sensing waveform. By using $x^{H}Ax=\mathrm{Tr}\left(Axx^{H}\right)$, the quadratic terms in Equations (20)–(23) can be expressed as affine trace functions of the lifted variables. For compactness, we define

(29) $$\begin{array}{c} M_{1,k}≜\sigma_{t}^{2}A^{H}uu^{H}A, \end{array}$$

(30) $$\begin{array}{c} M_{2,k}≜\sigma_{t}^{2}\lambda \mathrm{diag}\left\{A^{H}uu^{H}A\right\}, \end{array}$$

(31) $$\begin{array}{c} M_{3,k}≜\mathrm{diag}\left\{h_{k}h_{k}^{H}\right\}, \end{array}$$

(32) $$\begin{array}{c} M_{4,k}≜\lambda \mathrm{diag}\left\{M_{a}\right\}+M_{a}, \end{array}$$

(33) $$\begin{array}{c} M_{a,k}≜\sigma_{t}^{2}G^{H}\Phi^{H}h_{r,t}h_{r,t}^{H}\Phi \Phi^{H}h_{r,t}h_{r,t}^{H}\Phi G+G^{H}\Phi^{H}\Phi G. \end{array}$$

By applying the above definitions, the problem in Equations (20)–(23) can be equivalently reformulated as a lifted matrix optimization problem with the rank-one constraints $\mathrm{rank}\{Q_{i}\}=1,\forall i$. Given the non-convexity of these constraints, semidefinite relaxation (SDR) is employed by omitting them. The resulting relaxed transmit beamforming problem is then expressed as

(34) $$\begin{array}{cc} \max_{{\left\{Q_{i}\right\}}_{i=1}^{K},\;S}\;\; & \frac{\mathrm{Tr}\left(M_{1}S\right)}{\mathrm{Tr}\left(M_{2}S\right)+c_{u}} \end{array}$$

(35) $$\begin{array}{cc} s.t.\;\; & \left(1+\lambda \right)\mathrm{Tr}\left(S\right)\leq P_{\mathrm{BS}}, \end{array}$$

(36) $$\begin{array}{cc} \; & \left(1+\Gamma_{k}^{-1}\right)h_{k}^{H}Q_{k}h_{k}\geq h_{k}^{H}Sh_{k}+\lambda \mathrm{Tr}\left(M_{3,k}S\right)+c_{k},\;\forall k, \end{array}$$

(37) $$\begin{array}{cc} \; & \mathrm{Tr}\left(M_{4}S\right)\leq P_{\mathrm{RIS}}-c_{p}, \end{array}$$

(38) $$\begin{array}{cc} \; & Q_{i}⪰0,\;i=1,\ldots ,K, \end{array}$$

(39) $$\begin{array}{cc} \; & S⪰0, \end{array}$$

(40) $$\begin{array}{cc} \; & S-\sum_{i=1}^{K}Q_{i}⪰0. \end{array}$$

Although the SDR reformulation yields a convex feasible region for Equations (34)–(40), its objective function remains a linear fractional function. Therefore, the Charnes–Cooper transformation is employed by introducing the auxiliary variable $t≜\frac{1}{\mathrm{Tr}\left(M_{2}S\right)+c_{u}}$ and the scaled variables $\hat{S}≜tS,\;{\hat{Q}}_{i}≜tQ_{i},\;i=1,\ldots ,K.$ Using this variable transformation, Equations (34)–(40) can be equivalently reformulated as

(41) $$\begin{array}{cc} \max_{{\left\{{\hat{Q}}_{i}\right\}}_{i=1}^{K},\;\hat{S},\;t}\;\; & \mathrm{Tr}\left(M_{1}\hat{S}\right) \end{array}$$

(42) $$\begin{array}{cc} s.t.\;\; & \mathrm{Tr}\left(M_{2}\hat{S}\right)+tc_{u}=1, \end{array}$$

(43) $$\begin{array}{cc} \; & \left(1+\lambda \right)\mathrm{Tr}\left(\hat{S}\right)\leq tP_{\mathrm{BS}}, \end{array}$$

(44) $$\begin{array}{cc} \; & \mathrm{Tr}\left(M_{4}\hat{S}\right)\leq t\left(P_{\mathrm{RIS}}-c_{p}\right), \end{array}$$

(45) $$\begin{array}{cc} \; & \left(1+\Gamma_{k}^{-1}\right)h_{k}^{H}{\hat{Q}}_{k}h_{k}\geq h_{k}^{H}\hat{S}h_{k}+\lambda \mathrm{Tr}\left(M_{3,k}\hat{S}\right)+tc_{k},\;\forall k, \end{array}$$

(46) $$\begin{array}{cc} \; & {\hat{Q}}_{i}⪰0,\;i=1,\ldots ,K, \end{array}$$

(47) $$\begin{array}{cc} \; & \hat{S}⪰0, \end{array}$$

(48) $$\begin{array}{cc} \; & \hat{S}-\sum_{i=1}^{K}{\hat{Q}}_{i}⪰0, \end{array}$$

(49) $$\begin{array}{cc} \; & t>0. \end{array}$$

The SDP formulated in Equations (41)–(49) is convex and can be efficiently solved using standard convex optimization solvers, such as CVX. Since the Charnes–Cooper transformation introduces the scaling variable *t*, the matrix variables in the original scale need to be recovered after solving Equations (41)–(49). Let ${\hat{S}}^{⋆}$, ${\hat{Q}}_{i}^{⋆}$, and $t^{⋆}$ denote the optimal solutions of Equations (41)–(49). Then, the corresponding unscaled matrix variables are recovered as

(50) $$\begin{array}{c} S^{⋆}\;=\frac{{\hat{S}}^{⋆}}{t^{⋆}}, \end{array}$$

(51) $$\begin{array}{c} Q_{i}^{⋆}\;=\frac{{\hat{Q}}_{i}^{⋆}}{t^{⋆}},\;i=1,\ldots ,K. \end{array}$$

If the obtained $Q_{i}^{⋆}$ satisfies the rank-one condition, the communication beamforming vector for the *i*-th user can be recovered. Otherwise, a feasible approximate beamforming vector can be obtained using Gaussian randomization or the principal eigenvector method. For the rank-one case, the communication beamforming vector can be recovered as

(52) $$w_{i}=\frac{Q_{i}^{⋆}h_{i}}{\sqrt{h_{i}^{H}Q_{i}^{⋆}h_{i}}},\;i=1,\ldots ,K.$$

For the radar beamforming part, the residual radar covariance matrix is constructed by subtracting the communication beamforming covariance from the total transmit covariance. The radar beamforming matrix is then obtained from this residual covariance matrix via Cholesky decomposition or eigenvalue decomposition, i.e.,

(53) $$W_{r}W_{r}^{H}=S^{⋆}-\sum_{i=1}^{K}w_{i}w_{i}^{H}.$$

Finally, the complete BS transmit beamforming matrix is constructed by stacking the recovered communication beamforming vectors and the radar beamforming matrix, namely, $W=[w_{1},\ldots ,w_{K},W_{r}]$.

### 3.3. Reflection Coefficient Design

Given the receive filter u and the BS transmit beamforming matrix W, the optimization subproblem with respect to the active RIS reflection coefficient vector ϕ can be written as:

(54) $$\begin{array}{cc} \max_{\phi }\;\; & \frac{f_{1}\left(\phi \right)}{f_{2}\left(\phi \right)} \end{array}$$

(55) $$\begin{array}{cc} s.t.\;\; & \gamma_{k}\geq \Gamma_{k},\;\forall k, \end{array}$$

(56) $$\begin{array}{cc} \; & P_{\mathrm{ris}}\leq P_{\mathrm{RIS}}, \end{array}$$

(57) $$\begin{array}{cc} \; & a_{m}\leq a_{\max},\;\forall m. \end{array}$$

where $f_{1}\left(\phi \right)$ and $f_{2}\left(\phi \right)$ are defined as follows:

(58) $$\begin{array}{c} f_{1}\left(\phi \right)\;≜\sigma_{t}^{2}u^{H}AWW^{H}A^{H}u, \end{array}$$

(59) $$\begin{array}{c} f_{2}\left(\phi \right)\;≜u^{H}\left(\sigma_{t}^{2}AR_{b}A^{H}+\sigma_{t}^{2}\sigma_{s}^{2}BB^{H}+\sigma_{s}^{2}PP^{H}+\sigma_{r}^{2}I_{N}\right)u. \end{array}$$

Since the objective function in Equation (54) is fractional, the Dinkelbach transformation is applied. By introducing an auxiliary variable η, the fractional objective can be transformed into the following subtractive form:

(60) $$\max_{\phi }\;f_{1}\left(\phi \right)-\eta f_{2}\left(\phi \right).$$

At the *ℓ*-th iteration, the Dinkelbach auxiliary variable η is updated as

(61) $$\eta^{\left(\ell \right)}=\frac{f_{1}\left(\phi^{\left(\ell -1\right)}\right)}{f_{2}\left(\phi^{\left(\ell -1\right)}\right)}.$$

Although Equation (60) removes the fractional structure, the active RIS reflection coefficient vector is still implicitly contained in the cascaded channel matrices. To make the dependence on ϕ explicit, we use $\Phi^{H}h_{r,t}=\mathrm{diag}\left\{h_{r,t}\right\}\phi^{*}$. Then, by defining $\tilde{G}≜G^{H}\mathrm{diag}\left\{h_{r,t}\right\}$ and $\psi ≜\phi^{*}$, the target-related cascaded channel matrix can be expressed as $A=\tilde{G}\psi \psi^{H}{\tilde{G}}^{H}$, $f_{1}\;\left(\phi \right)$ can be rewritten as

(62) $$\begin{array}{cc} f_{1}\left(\phi \right) & =\sigma_{t}^{2}u^{H}\tilde{G}\psi \psi^{H}{\tilde{G}}^{H}WW^{H}\tilde{G}\psi \psi^{H}{\tilde{G}}^{H}u, \end{array}$$

(63) $$\begin{array}{cc} \; & =\sigma_{t}^{2}\mathrm{Tr}\left({\tilde{G}}^{H}uu^{H}\tilde{G}\psi \psi^{H}{\tilde{G}}^{H}WW^{H}\tilde{G}\psi \psi^{H}\right), \end{array}$$

(64) $$\begin{array}{cc} \; & =\sigma_{t}^{2}{\mathrm{vec}}^{H}\left\{\psi \psi^{H}\right\}\left[\left({\tilde{G}}^{T}W^{*}W^{T}{\tilde{G}}^{*}\right)⊗\left({\tilde{G}}^{H}uu^{H}\tilde{G}\right)\right]\mathrm{vec}\left\{\psi \psi^{H}\right\}, \end{array}$$

(65) $$\begin{array}{cc} \; & =z^{H}Cz. \end{array}$$

The transition from Equation (63) to Equation (64) is obtained using the vectorization identity $\mathrm{Tr}\left(ABCD\right)={\mathrm{vec}}^{H}\left\{D^{H}\right\}\left(C^{T}⊗A\right)\mathrm{vec}\left\{B\right\}$. In Equation (65), the auxiliary vector and matrix are defined as $z≜\mathrm{vec}\left\{\psi \psi^{H}\right\}=\phi ⊗\phi^{*}$ and $C≜\sigma_{t}^{2}\left({\tilde{G}}^{T}W^{*}W^{T}{\tilde{G}}^{*}\right)⊗\left({\tilde{G}}^{H}uu^{H}\tilde{G}\right)$. Similarly, the denominator function $f_{2}\left(\phi \right)$ contains the dynamic thermal noise introduced by the active RIS and the distortion noise caused by BS transmitter HWIs. Following the same vectorization procedure used in Equations (63)–(65), $f_{2}\left(\phi \right)$ in Equation (59) can be represented as

(66) $$f_{2}\left(\phi \right)=z^{H}C_{1}z+z^{H}Dz+\phi^{H}E\phi +\sigma_{r}^{2}{∥u∥}_{2}^{2},$$

where $C_{1}$, D, and E are the corresponding auxiliary matrices, given by

(67) $$\begin{array}{c} C_{1}\;≜\sigma_{t}^{2}\lambda \left[\left({\tilde{G}}^{T}\mathrm{diag}\left\{WW^{H}\right\}{\tilde{G}}^{*}\right)⊗\left({\tilde{G}}^{H}uu^{H}\tilde{G}\right)\right], \end{array}$$

(68) $$\begin{array}{c} D\;≜\sigma_{t}^{2}\sigma_{s}^{2}\left[\left(\mathrm{diag}\left\{h_{r,t}\right\}\mathrm{diag}\left\{h_{r,t}^{*}\right\}\right)⊗\left({\tilde{G}}^{H}uu^{H}\tilde{G}\right)\right], \end{array}$$

(69) $$\begin{array}{c} E\;≜\sigma_{s}^{2}{\left(\mathrm{diag}\left\{Gu\right\}\right)}^{H}\mathrm{diag}\left\{Gu\right\}. \end{array}$$

Since E is a real diagonal positive semidefinite matrix, we have $\phi^{H}E\phi =\psi^{H}E\psi$. Therefore, Equation (66) can be equivalently expressed as

(70) $$f_{2}\left(\phi \right)=z^{H}C_{1}z+z^{H}Dz+\psi^{H}E\psi +\sigma_{r}^{2}{∥u∥}_{2}^{2}.$$

Substituting Equations (65) and (70) into Equation (60), the optimization subproblem with respect to the active RIS reflection coefficient vector can be reformulated as

(71) $$\min_{\psi }\;z^{H}Fz+\eta \psi^{H}E\psi +c_{1},$$

where the equivalent weighting matrix F and the constant $c_{1}$ are defined as $F≜\eta C_{1}+\eta D-C$ and $c_{1}≜\sigma_{r}^{2}\eta {∥u∥}_{2}^{2}$. Although the variable extraction and vectorization make the dependence on ψ explicit, Equation (71) remains difficult to solve because $z=\mathrm{vec}\{\psi \psi^{H}\}$, and thus $z^{H}Fz$ is quartic with respect to ψ. To obtain a tractable surrogate problem, the MM method is applied. At the *n*-th MM iteration, a second-order Taylor-based upper bound of $z^{H}Fz$ at $z_{n}$ is given by

(72) zHFz≤λfzHz+2RezHF−λfIzn+znHλfI−Fzn,

where $\lambda_{f}$ denotes the largest eigenvalue of F. Considering the amplitude constraint $a_{m}\leq a_{\max}$, the term $z^{H}z$ can be further bounded as

(73) $$\begin{array}{cc} z^{H}z & ={\left(\psi^{*}⊗\psi \right)}^{H}\left(\psi^{*}⊗\psi \right)\;\\ \; & ={\left(\phi ⊗\phi^{*}\right)}^{H}\left(\phi ⊗\phi^{*}\right)\\ \; & \leq M^{2}a_{\max}^{4}. \end{array}$$

Substituting the upper bound in Equation (73) into Equation (72), the quartic term can be further bounded as

(74) zHFz≤RezHf+c2,

(75) =ReψHFeψ+c2,

where $c_{2}≜\lambda_{f}M^{2}a_{\max}^{4}+z_{n}^{H}\left(\lambda_{f}I-F\right)z_{n}$ is a constant independent of the optimization variable, $f≜2\left(F-\lambda_{f}I\right)z_{n}$ is the linear coefficient vector determined by the current iteration point $z_{n}$, and $F_{e}$ denotes the matrix form of f, satisfying $f≜\mathrm{vec}\{F_{e}\}$. Since Re{ψHFeψ} is still a non-convex quadratic term with respect to ψ, the complex variable is further transformed into an equivalent real-valued form. Define ψ¯≜ReψT,ImψTT and construct the block real-valued matrix F¯e≜Re{Fe}−Im{Fe}Im{Fe}Re{Fe}. Subsequently, a second-order Taylor expansion of ${\bar{\psi }}^{T}{\bar{F}}_{e}\bar{\psi }$ is performed at the current local feasible point ${\bar{\psi }}_{n}$. A convex upper-bound surrogate of ${\bar{\psi }}^{T}{\bar{F}}_{e}\bar{\psi }$ can be constructed as follows:

$$\begin{array}{cc} {\bar{\psi }}^{T}{\bar{F}}_{e}\bar{\psi } & \leq {\bar{\psi }}_{n}^{T}{\bar{F}}_{e}{\bar{\psi }}_{n}+{\bar{\psi }}_{n}^{T}\left({\bar{F}}_{e}+{\bar{F}}_{e}^{T}\right)\left(\bar{\psi }-{\bar{\psi }}_{n}\right) \end{array}$$

(76) $$\begin{array}{cc} \; & \;+\frac{{\tilde{\lambda }}_{f}}{2}{\left(\bar{\psi }-{\bar{\psi }}_{n}\right)}^{T}\left(\bar{\psi }-{\bar{\psi }}_{n}\right), \end{array}$$

(77) =λ˜f2ψHψ+ReψHef+c3,

where ${\tilde{\lambda }}_{f}$ denotes the largest eigenvalue of the Hessian matrix $\left({\bar{F}}_{e}+{\bar{F}}_{e}^{T}\right)$, $e_{f}≜U\left({\bar{F}}_{e}+{\bar{F}}_{e}^{T}-{\tilde{\lambda }}_{f}I\right){\bar{\psi }}_{n}$ denotes the linear coefficient vector constructed from the current iteration point, with $U≜[I_{M},jI_{M}]$, and $c_{3}≜-{\bar{\psi }}_{n}^{T}{\bar{F}}_{e}{\bar{\psi }}_{n}+\frac{{\tilde{\lambda }}_{f}}{2}{\bar{\psi }}_{n}^{T}{\bar{\psi }}_{n}$ is a constant independent of the optimization variable. Combining Equations (74), (75) and (77), the quartic term in Equation (71) admits the following quadratic upper bound:

(78) zHFz≤λ˜f2ψHψ+ReψHef+c2+c3.

Therefore, the optimization with respect to ψ in Equation (71) can be further transformed into the following surrogate subproblem:

(79) minψψHEeψ+ReψHef,

where $E_{e}$ denotes the coefficient matrix in the surrogate objective and is defined as

(80) $$E_{e}≜\eta E+\frac{{\tilde{\lambda }}_{f}}{2}I_{M}.$$

However, the active RIS power constraint remains non-convex due to the high-order coupling terms associated with the reflection vector ψ. To facilitate subsequent convexification, we represent the variable ψ in the power function P(ψ), and rewrite it as

(81) $$P\left(\psi \right)=z^{H}Jz+\psi^{H}K\psi ,$$

where the auxiliary matrices in Equation (81) are defined as

$$\begin{array}{c} J_{e}\;≜\sigma_{t}^{2}{\left(\mathrm{diag}\left\{h_{r,t}^{*}\right\}GR_{x}G^{H}\mathrm{diag}\left\{h_{r,t}\right\}\right)}^{T}⊗J_{e} \end{array}$$

(82) $$\begin{array}{cc} \; & \;+\sigma_{t}^{2}\sigma_{s}^{2}\left(J_{e}⊗J_{e}\right), \end{array}$$

(83) $$\begin{array}{c} J_{e}\;≜\mathrm{diag}\left\{h_{r,t}^{*}\right\}\mathrm{diag}\left\{h_{r,t}\right\}, \end{array}$$

(84) $$\begin{array}{c} K_{e}\;≜\mathrm{diag}\left\{d\right\}+2\sigma_{s}^{2}I_{M}, \end{array}$$

(85) $$\begin{array}{c} d_{e}\;≜\mathrm{diag}\left\{D_{1}\right\}, \end{array}$$

(86) $$\begin{array}{c} D_{1}\;≜GR_{x}G^{H}. \end{array}$$

Based on Equation (81), the active RIS power function still contains the quartic coupling term $z^{H}Jz$. To handle this term, the same MM-based upper-bound construction used for the objective function is adopted. At the *n*-th MM iteration, an upper bound of $z^{H}Jz$ can be constructed as

(87) zHJz≤RezHp+c4=ReψHPeψ+c4,

(88) =ψ¯TP¯eψ¯+c4≤λp2ψHψ+ReψHep+c5,

where $p≜2\left(J-\lambda_{j}I\right)z_{n}$ denotes the linearized coefficient vector determined by the current iteration point $z_{n}$, with $\lambda_{j}≜\mathrm{Tr}\left(J\right)$. The matrix $P_{e}$ denotes the matrix form of p, satisfying $p≜\mathrm{vec}\{P_{e}\}$. $c_{4}≜\lambda_{j}M^{2}a_{\max}^{4}+z_{n}^{H}\left(\lambda_{j}I-J\right)z_{n}$ is a constant independent of the optimization variable. P¯e≜Re{Pe}−Im{Pe}Im{Pe}Re{Pe} denotes the real-valued equivalent representation of $P_{e}$, $\lambda_{p}$ denotes the largest eigenvalue of the Hessian matrix $\left({\bar{P}}_{e}+{\bar{P}}_{e}^{T}\right)$, $e_{p}≜U\left({\bar{P}}_{e}+{\bar{P}}_{e}^{T}-\lambda_{p}I\right){\bar{\psi }}_{n}$ is the linear coefficient vector constructed from the current iteration point, with $U≜[I_{M},jI_{M}]$, and $c_{5}≜-{\bar{\psi }}_{n}^{T}{\bar{P}}_{e}{\bar{\psi }}_{n}+\frac{\lambda_{p}}{2}{\bar{\psi }}_{n}^{T}{\bar{\psi }}_{n}+c_{4}$ are constants independent of the optimization variable. According to Equations (81), Equations (87) and (88), the active RIS power function can be upper-bounded as

(89) P(ψ)≤ψHKeψ+ReψHep+c5,

where $K_{e}$ denotes the equivalent coefficient matrix, defined as

(90) $$K_{e}≜K+\frac{\lambda_{p}}{2}I_{M},$$

where ${\bar{h}}_{k}\left(\psi \right)$ denotes the equivalent channel of user *k*:

(91) $${\bar{h}}_{k}\left(\psi \right)≜h_{b,k}+G^{H}\mathrm{diag}\left\{h_{r,k}\right\}\psi .$$

The desired signal term and the stacked interference-plus-noise vector of user *k* are respectively given by

(92) $$\begin{array}{c} e_{a,k}\left(\psi \right)\;≜a_{k,k}+b_{k,k}^{H}\psi , \end{array}$$

(93) $$\begin{array}{c} b_{e,k}\left(\psi \right)\;≜\left[\begin{array}{c} a_{k}+B_{k}^{H}\psi \\ R_{b}^{\frac{1}{2}}{\bar{h}}_{k}\left(\psi \right)\\ \sigma_{s}\mathrm{diag}\left\{h_{r,k}\right\}\psi \\ \sigma_{k} \end{array}\right], \end{array}$$

where $a_{k,i}$ and $b_{k,i}$ denote the scalar and vector terms associated with the *i*-th transmit beam and the effective channel of user *k*, respectively. For subsequent derivations, these terms are collected into the vector $a_{k}$ and the matrix $B_{k}$, which are defined as follows:

(94) $$\begin{array}{c} a_{k,i}\;≜w_{i}^{H}h_{b,k}, \end{array}$$

(95) $$\begin{array}{c} b_{k,i}\;≜\mathrm{diag}\left\{h_{r,k}^{*}\right\}Gw_{i}, \end{array}$$

(96) $$\begin{array}{c} a_{k}\;≜{\left[a_{k,1},\ldots ,a_{k,K+N}\right]}^{T}, \end{array}$$

(97) $$\begin{array}{c} B_{k}\;≜\left[b_{k,1},\ldots ,b_{k,K+N}\right]. \end{array}$$

Therefore, under appropriate phase normalization, the QoS constraint of each communication user can be further cast into the following second-order cone (SOC) form:

(98) $$\sqrt{1+\Gamma_{k}}\left|e_{a,k}\left(\psi \right)\right|\geq \sqrt{\Gamma_{k}}{∥b_{e,k}\left(\psi \right)∥}_{2}.\;\forall k.$$

In summary, for fixed receive filter u and BS transmit beamforming matrix W, the original active RIS reflection coefficient optimization subproblem is reformulated into the following convex surrogate subproblem after the Dinkelbach transformation, explicit variable representation, vectorization, MM-based upper-bound approximation, and SOC constraint transformation:

(99) minψψHEeψ+ReψHef

(100) $$\begin{array}{cc} s.t.\;\; & a_{m}\leq a_{\max},\;\forall m, \end{array}$$

(101) ψHKeψ+ReψHep≤PRIS−c5,

(102) $$\begin{array}{cc} \; & \sqrt{1+\Gamma_{k}}\left|e_{a,k}\left(\psi \right)\right|\geq \sqrt{\Gamma_{k}}{∥b_{e,k}\left(\psi \right)∥}_{2}.\;\forall k. \end{array}$$

The resulting convex surrogate subproblem in Equations (99)–(102) can be solved by CVX.

### 3.4. Summary

The computational complexity of the proposed AO-based algorithm mainly arises from the updates of the receive filter, the BS transmit beamforming matrix, and the active RIS reflection coefficient vector. The convex surrogate subproblems are solved using interior-point methods. For the receive filter update, an eigenvalue decomposition of an N×N matrix is required, leading to a complexity of $O(N^{3})$. For the BS transmit beamforming design, solving the standard SDP problem in the worst case and recovering the radar beamforming matrix through matrix decomposition require complexities of $O\left({\left(K+1\right)}^{3.5}N^{6.5}\right)$ and $O(N^{3})$, respectively. For the optimization of the active RIS reflection coefficient vector ψ, the update of the Dinkelbach parameter and the computation of the auxiliary matrices $E_{e}$ and $K_{e}$ require $O(M^{2})$. The convex surrogate subproblem with SOC constraints has a complexity of $O(M^{3.5})$. Denoting the number of inner MM iterations by $I_{\mathrm{in}}$, the complexity of the reflection coefficient design module is $O(I_{\mathrm{in}}M^{3.5})$. In addition, updating the equivalent cascaded channel matrix requires $O(N^{2}M)$. Therefore, by denoting the number of outer AO iterations as $I_{\mathrm{out}}$ and ignoring lower-order terms and constant factors, the overall computational complexity of the proposed joint beamforming and active RIS reflection design algorithm can be expressed as $O\left(I_{\mathrm{out}}\left({\left(K+1\right)}^{3.5}N^{6.5}+I_{\mathrm{in}}M^{3.5}+N^{2}M\right)\right)$. It should be emphasized that the above complexity expression corresponds to the worst-case theoretical asymptotic upper bound of the generic dense interior-point method.

## 4. Simulation Results

In this section, numerical simulations are conducted to evaluate the performance of the proposed active RIS-assisted ISAC joint beamforming and reflection design scheme in the presence of BS transmitter HWIs. All numerical results are obtained under the perfect global CSI assumption adopted in Section 2. The target AoA and AoD relative to the active RIS are also assumed to be perfectly known, and continuous and independent amplitude and phase control is adopted for the active RIS coefficients. Accordingly, the simulations are intended to characterize the effects of BS transmitter HWIs and the interaction between BS transmitter distortion noise and the active RIS amplification mechanism and power constraint under ideal-CSI, ideal-angular-information, and continuous-control benchmarks.

The numbers of BS antennas, active RIS elements, and communication users are set to N=16, M=32, and K=4, respectively. The noise powers are set to $\sigma_{k}^{2}=\sigma_{s}^{2}=\sigma_{r}^{2}=-80\;\mathrm{dBm}$ for all *k*. The maximum power budgets of the BS and the active RIS are set to $P_{\mathrm{BS}}=40\;\mathrm{dBm}$ and $P_{\mathrm{RIS}}=20\;\mathrm{dBm}$, respectively. The target RCS is normalized to $\sigma_{t}^{2}=1$. The distances of the BS–RIS, RIS–target, BS–user, and RIS–user links are set to $d_{\mathrm{br}}=50\;m$, $d_{\mathrm{rt}}=5\;m$, $d_{\mathrm{bu}}=50\;m$, and $d_{\mathrm{ru}}=10\;m$, respectively. A distance-dependent path-loss model is adopted, with the path-loss exponents of these four links set to 2.2, 2.2, 3.5, and 2.3, respectively [30]. The BS–RIS link follows a Rician fading model with a Rician factor of κ=5dB, whereas the BS–user and RIS–user links follow Rayleigh fading models. The RIS–target link is modeled as a LoS channel. For ease of analysis, all users are assumed to have the same SINR threshold, i.e., $\Gamma_{k}=\Gamma$. The maximum amplification factor of each active RIS element is set to $a_{\max}\in \left\{4,8\right\}$. Unless otherwise specified, the BS transmitter HWI coefficient is set to λ=0.06. Unless otherwise specified, the numerical observations reported in this section should be interpreted within the considered system configuration, including the blocked direct link, propagation geometry, power budgets, active RIS amplification limits, and adopted channel and noise models.

**Figure 2:**
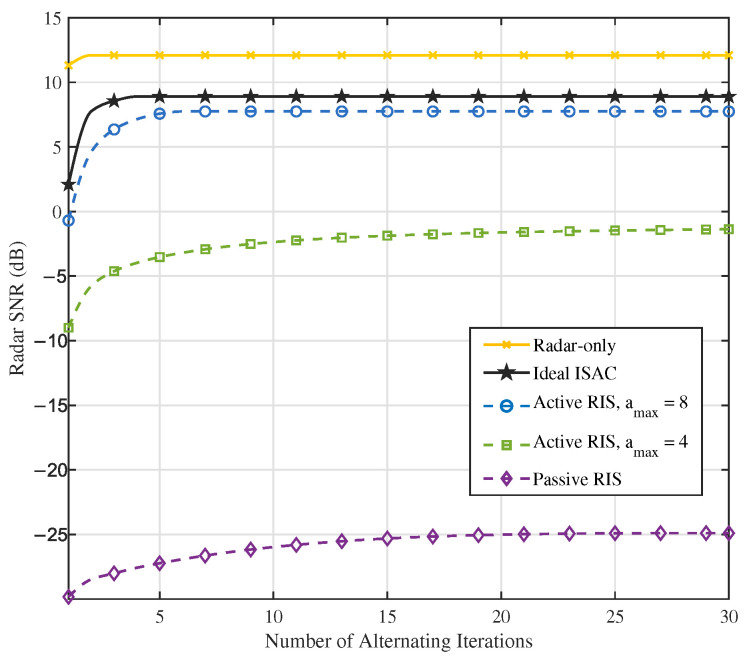
Radar output SNR versus the number of alternating iterations.

The convergence performance of the proposed AO algorithm under the considered ideal-CSI benchmark is shown in Figure 2. Under the baseline system configuration, the radar output SNR of all considered schemes increases rapidly during the first few iterations and then gradually approaches a stable value. This observation indicates that the proposed iterative framework exhibits stable numerical convergence for the considered perfect-CSI optimization problem. The ideal ISAC and active RIS-assisted schemes converge within approximately 5–10 iterations and achieve higher converged SNRs than the passive RIS scheme. This performance advantage is mainly attributed to the amplitude amplification capability of the active RIS, which enhances the effective echo strength of the cascaded sensing link. In addition, the radar-only scheme is not constrained by communication QoS requirements and therefore achieves the highest radar output SNR, serving as a reference benchmark for sensing performance.

**Figure 3:**
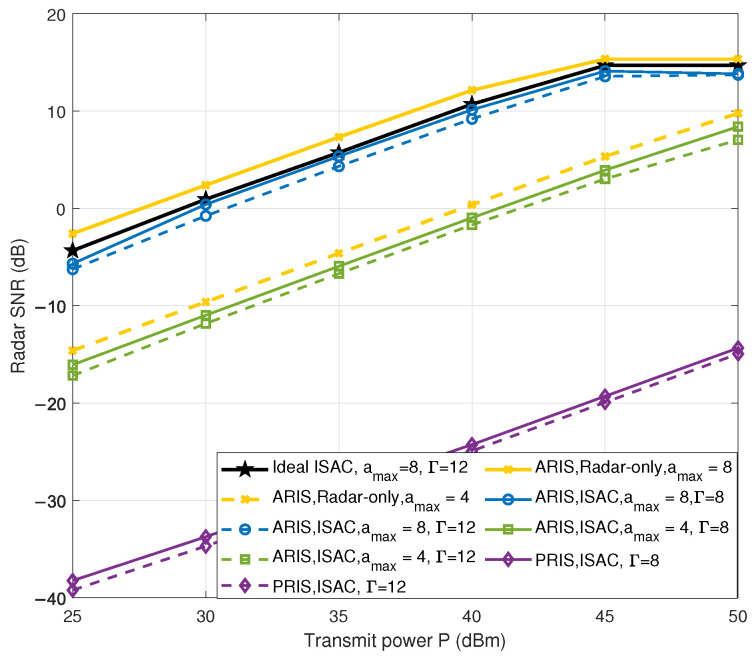
Radar output SNR versus the BS transmit power budget.

The radar output SNR versus the BS transmit power *P* is shown in Figure 3. As *P* increases, all schemes achieve higher radar output SNRs, although the improvement becomes less pronounced in the high-power region, e.g., P>40dBm. This is because increasing the transmit power strengthens the desired radar echo while also increasing the signal-dependent distortion noise induced by BS transmitter HWIs. Meanwhile, the active RIS power budget limits the available amplification gain, and the active RIS amplification noise also affects the cascaded sensing link. Under the baseline configuration, when P=50dBm, the ARIS-ISAC scheme with $a_{\max}=8$ and Γ=8 achieves a radar output SNR of approximately 12dB, whereas the PRIS-ISAC benchmark achieves approximately −20dB. This numerical difference illustrates the benefit of active amplification for the considered blocked cascaded sensing link. However, the magnitude of the observed gain depends on the active RIS power budget, amplification-factor limit, propagation geometry, path-loss characteristics, and noise conditions. In addition, reducing $a_{\max}$ from 8 to 4 decreases the radar output SNR, indicating that the sensing performance is sensitive to the available active reflection gain. The ideal-hardware benchmark achieves a higher radar output SNR, illustrating the performance degradation caused by BS transmitter distortion noise under the considered settings.

**Figure 4:**
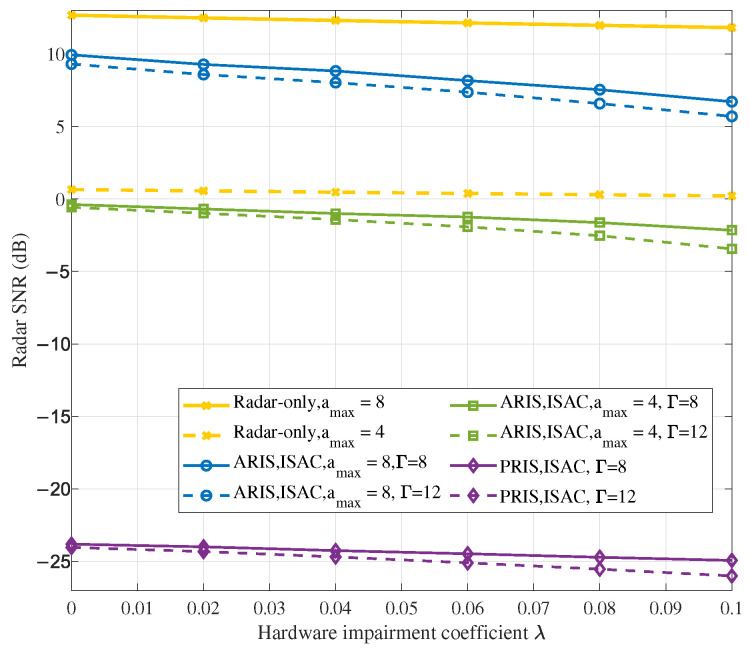
Radar output SNR versus the transmitter hardware impairment coefficient.

The effect of BS transmitter HWIs on radar sensing performance is shown in Figure 4. As the hardware impairment coefficient λ increases, all schemes exhibit a decreasing radar output SNR. The degradation can be attributed to the increased distortion noise induced by BS transmitter HWIs, which propagates through the active RIS-assisted cascaded sensing link and weakens the effective radar echo quality. Under the same hardware impairment level, ARIS-ISAC with $a_{\max}=8$ consistently achieves a higher radar output SNR than that with $a_{\max}=4$, confirming the effectiveness of active reflection gain in enhancing the cascaded sensing link. It is further observed that the active RIS reflection amplitude limit affects the sensitivity of radar sensing performance to BS transmitter HWIs. However, from the variation trend of the curve slopes, it can be further observed that as the hardware impairment coefficient λ increases, the decrease in radar output SNR becomes more pronounced. Specifically, when Γ=12, the radar output SNR decreases from approximately −0.5dB to −3.5dB for $a_{\max}=4$, corresponding to an SNR loss of approximately 3dB. In contrast, for $a_{\max}=8$, the radar output SNR decreases from approximately 9.3dB to 5.6dB, corresponding to a larger SNR loss of approximately 3.7dB. Therefore, although a larger reflection amplitude limit provides a higher absolute radar output SNR, its descending trend becomes more evident as λ increases, suggesting that the system becomes more sensitive to variations in the hardware impairment coefficient. Under non-ideal hardware conditions, the design of the active RIS reflection amplitude limit should strike a balance between active reflection gain and hardware-impairment sensitivity. Accordingly, the active RIS reflection amplitude should be properly configured according to the hardware impairment level, so as to enhance the cascaded sensing link gain while mitigating the impact of hardware distortion propagation on radar performance.

**Figure 5:**
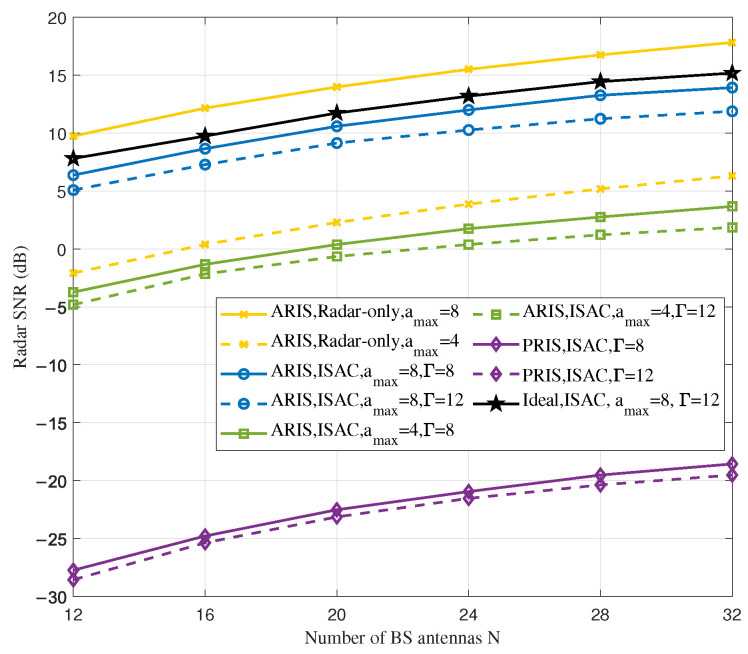
Radar output SNR versus the number of BS antennas.

The influence of the number of BS antennas *N* on radar sensing performance is shown in Figure 5. As *N* increases from 12 to 32, all schemes achieve higher radar output SNRs due to the additional spatial degrees of freedom and array gain. However, the improvement becomes less pronounced when N>24, since the achievable gain is limited by BS transmitter HWIs, the active RIS power budget, and communication QoS constraints. The gap between the ideal hardware benchmark and ARIS-ISAC also increases with *N*, showing that transmitter-hardware-impairment-induced distortion weakens part of the antenna-array gain. Moreover, a higher QoS threshold leads to a lower radar output SNR, reflecting the communication–sensing tradeoff. Compared with PRIS-ISAC, ARIS-ISAC consistently achieves higher SNRs, confirming the benefit of active reflection gain in compensating for the blocked cascaded link loss.

**Figure 6:**
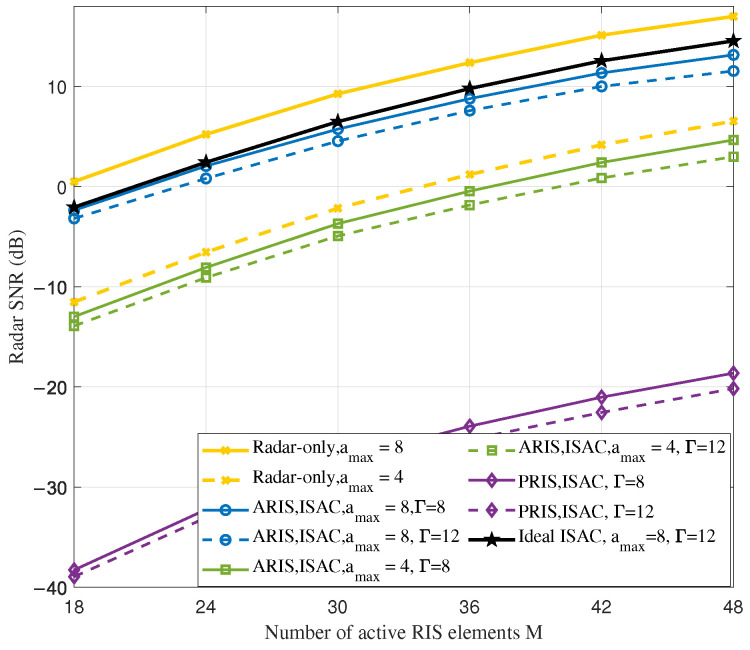
Radar output SNR versus the number of active RIS elements.

The effect of the RIS array size *M* on radar sensing performance is shown in Figure 6. As *M* increases from 18 to 48, all schemes achieve higher radar output SNRs, since more RIS reflecting elements provide additional spatial degrees of freedom and enhance the cascaded sensing link. However, the SNR improvement gradually becomes less pronounced for large *M*, reflecting a diminishing marginal gain under the constraints of BS transmitter HWIs, active RIS power budget, and communication QoS requirements. When M=48, ARIS-ISAC ($a_{\max}=8$, Γ=8) achieves approximately 12dB, whereas PRIS-ISAC achieves approximately −22dB, showing the benefit of active reflection gain. Moreover, the SNR gap between $a_{\max}=8$ and $a_{\max}=4$ becomes larger as *M* increases, which indicates that a larger RIS aperture can better exploit the active reflection amplitude limit to enhance radar sensing performance.

**Figure 7:**
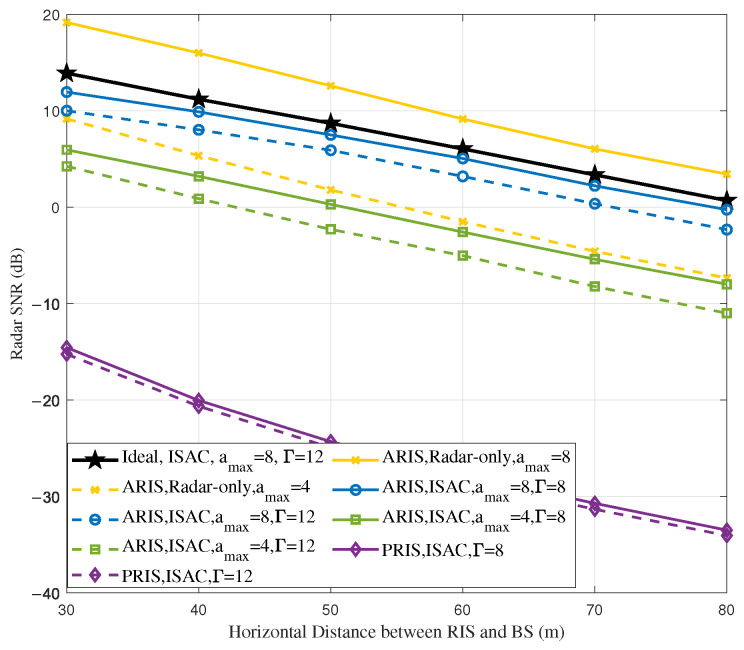
Radar output SNR versus the horizontal distance between the RIS and the BS.

The impact of the RIS–BS horizontal distance on the radar output SNR is shown in Figure 7 under non-ideal hardware conditions. As the distance increases, all schemes exhibit a decreasing radar output SNR due to the increased path loss of the BS–RIS link. Since the radar echo relies on the BS–RIS–target–RIS–BS round-trip cascaded sensing link, a longer RIS–BS distance weakens the target echo strength more significantly. When the distance is 30m, ARIS-ISAC ($a_{\max}=8$, Γ=8) achieves approximately 11.5dB, whereas PRIS-ISAC achieves approximately −15dB. Even when the distance increases to 80m, ARIS-ISAC still maintains approximately 0dB, while PRIS-ISAC remains at a much lower SNR level. This result shows that the active reflection gain of the active RIS can effectively enhance the echo strength over long-distance cascaded sensing links. Moreover, the gap between the ideal hardware benchmark and ARIS-ISAC reflects the performance degradation caused by BS transmitter HWIs, highlighting the necessity of joint beamforming optimization under practical hardware conditions.

**Figure 8:**
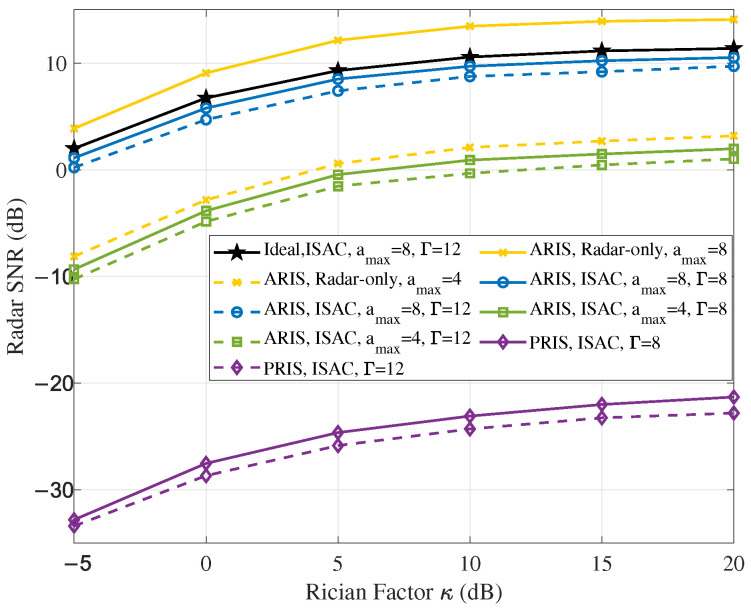
Radar output SNR versus the Rician factor.

The effect of the Rician factor κ on the radar output SNR is shown in Figure 8. As κ increases from −5dB to 20dB, all schemes achieve higher radar output SNRs and then gradually level off. This is mainly because a larger κ corresponds to a stronger LoS component and improved link directionality, which facilitates the coherent combination of the BS transmit beam and the RIS reflection beam. When κ becomes large, the SNR gain tends to saturate, since the performance is further limited by BS transmitter HWIs, the active RIS power budget, and communication QoS constraints. In a strong scattering environment, e.g., κ=−5dB, PRIS-ISAC achieves approximately −33dB, whereas ARIS-ISAC maintains approximately 1dB. This comparison shows that the active reflection gain of the active RIS can effectively enhance the cascaded sensing link under unfavorable channel conditions. Moreover, the ARIS-ISAC curve remains lower than the ideal hardware benchmark, confirming the performance degradation caused by BS transmitter HWIs.

**Figure 9:**
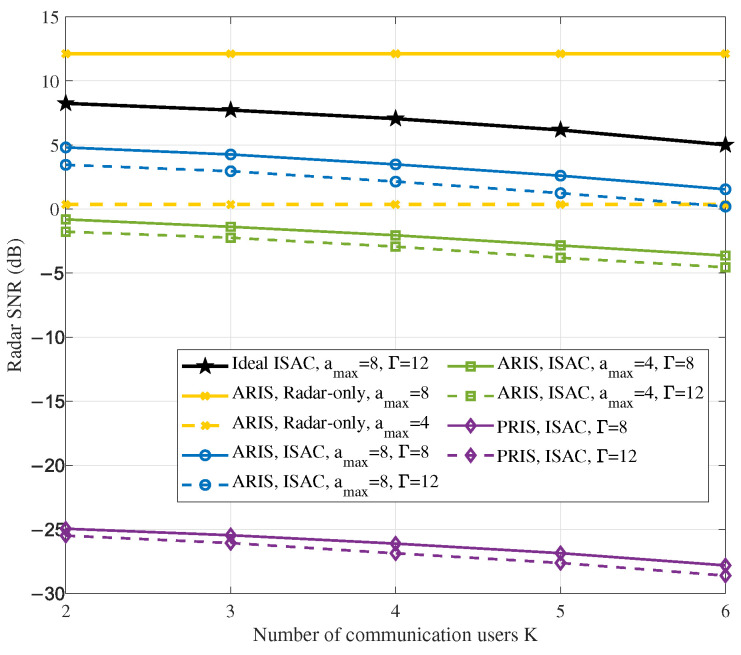
Radar output SNR versus the communication users.

The variation in radar sensing performance with the number of communication users is illustrated in Figure 9. Except for the radar-only schemes, the radar SNR of all ISAC schemes generally decreases as the number of users increases. This is because a larger number of communication users introduces more stringent QoS constraints and stronger multiuser interference, thereby requiring additional transmit power and spatial degrees of freedom to guarantee communication performance and reducing the resources available for radar sensing. For the same RIS configuration, the radar SNR achieved with Γ=8 dB is higher than that achieved with Γ=12 dB under the considered simulation settings, indicating that stricter communication SINR requirements may lead to a greater degradation in sensing performance. Moreover, the ARIS scheme with $a_{\max}=8$ achieves a higher radar SNR than that with $a_{\max}=4$, demonstrating the potential benefit of a stronger active reflection capability in compensating for the path loss of the double-hop cascaded channel. The ideal ISAC scheme also achieves a higher radar SNR than the corresponding scheme with transmitter hardware impairments, indicating that transmitter hardware distortion can degrade radar sensing performance. In addition, the ARIS schemes achieve noticeably higher radar SNR values than the PRIS schemes, highlighting the potential advantage of active RIS in enhancing the target echo and improving sensing performance. The radar-only curves remain nearly unchanged and can serve as useful reference benchmarks for radar performance under the corresponding parameter settings.

## 5. Conclusions

This paper investigated the joint beamforming optimization problem for an active RIS-assisted ISAC system with BS transmitter HWIs. Under the constraints of multiuser communication QoS, BS transmit power, active RIS power budget, and active RIS reflection amplitude, the radar output SNR was maximized by jointly optimizing the radar receive filter, the BS transmit beamforming matrix, and the active RIS reflection coefficients. The effects of distortion noise induced by BS transmitter HWIs, active RIS amplification noise, and the active RIS power constraint on both communication and sensing links were explicitly considered. To solve the resulting problem with a fractional objective function, coupled variables, and non-convex constraints, an alternating optimization-based iterative algorithm was proposed. Specifically, the radar receive filter was obtained in closed form based on the generalized Rayleigh quotient. The BS transmit beamforming subproblem was handled by SDR and the Charnes–Cooper transformation, while the active RIS reflection coefficient subproblem was solved by combining the Dinkelbach transformation and the MM method.

Under the considered simulation settings, numerical results showed that the proposed algorithm exhibited stable convergence behavior and that the active RIS-assisted scheme achieved a higher radar output SNR than the passive RIS-assisted benchmark by exploiting active reflection gain to enhance the RIS-assisted round-trip cascaded sensing link under direct-link blockage. The results also showed that transmitter HWIs and active RIS amplification noise degrade radar sensing performance, while a higher communication QoS threshold leads to a communication–sensing resource tradeoff. Furthermore, the results revealed a tradeoff between active reflection gain and hardware-impairment sensitivity: a larger active RIS reflection amplitude limit improves the absolute radar output SNR, whereas the corresponding SNR degradation becomes more evident as the hardware impairment coefficient increases. Therefore, the active RIS reflection amplitude should be configured according to the hardware impairment level rather than merely pursuing a larger reflection gain.

Several modeling assumptions and limitations should also be acknowledged. RSI after SIC at the FD BS is neglected, while perfect global CSI and perfect target angular information relative to the active RIS are assumed to be available at the BS. Accordingly, the present formulation and numerical results should be interpreted as ideal-CSI and ideal-angular-information benchmarks rather than as evidence of robustness against channel or angular uncertainty. In addition, the active RIS is modeled using continuously and independently adjustable complex coefficients, additive amplification noise, element-wise amplification limits, and an overall power budget. Thus, the reported results also represent an ideal continuous-control benchmark and do not explicitly account for finite-resolution control, circuit-dependent amplitude–phase coupling, or other RIS-side circuit HWIs. These modeling boundaries are adopted to maintain a focused investigation of BS transmitter HWIs and their interaction with the active RIS amplification mechanism. For future research, multiple extensions can be explored based on the current framework. First, the impact of non-negligible residual self-interference at the full-duplex BS, as well as robust joint beamforming design under channel state information uncertainty and target angular estimation errors, can be further investigated. Second, more practical active RIS response models can be incorporated, including quantized reflection coefficients with finite resolution, circuit-induced amplitude-phase coupling, and hardware-impaired active reflecting elements. In addition, more general BS transmitter hardware impairment models and multi-target sensing scenarios can also be considered as important extensions for active RIS-assisted ISAC systems.

## Data Availability

Data are contained within the article.
